# Relationship between heart rate variability and subclinical thyroid disorders of the Brazilian Longitudinal Study of Adult Health (ELSA-Brasil)

**DOI:** 10.1590/1414-431X20187704

**Published:** 2018-08-27

**Authors:** É.J.F. Peixoto de Miranda, R.A. Hoshi, M.S. Bittencourt, A.C. Goulart, I.S. Santos, A.R. Brunoni, M.F.H.S. Diniz, A.L.P. Ribeiro, E.M. Dantas, J.G. Mill, P.A. Lotufo, I.M. Benseñor

**Affiliations:** 1 Centro de Pesquisa Clínica, Hospital Universitário, Universidade de São Paulo, São Paulo, SP, Brasil; 2 Departamento de Ciências Fisiológicas, Universidade Federal do Espírito Santo, Vitória, ES, Brasil; 3 Departamento de Clínica Médica, Universidade Federal de Minas Gerais, Belo Horizonte, MG, Brasil; 4 Colegiado de Ciências Biológicas, Universidade Federal do Vale do São Francisco, Petrolina, PE, Brasil

**Keywords:** Subclinical hypothyroidism, Subclinical hyperthyroidism, Heart rate variability, Autonomic nervous system

## Abstract

The association between subclinical thyroid dysfunctions and autonomic modulation changes has been described by many studies with conflicting results. We aimed to analyze the association between subclinical hyperthyroidism (SCHyper), subclinical hypothyroidism (SCHypo), and heart rate variability (HRV) using the baseline from ELSA-Brasil. SCHyper and SCHypo were classified by use of medication to treat thyroid disorders, thyrotropin levels respectively above and under the reference range, and normal free thyroxine levels. For HRV, the participants underwent 10 min in supine position and the R-R intervals of the final 5 min were selected for analysis. We first used linear regression models to report crude data and then, multivariate adjustment for sociodemographic (age, sex, and race) and cardiovascular risk factors (hypertension, dyslipidemia, diabetes, smoking, body mass index, use of alcohol, and leisure physical activity) using the euthyroid group as reference. From 9270 subjects (median age, 50; interquartile range: 44–56), 8623 (93.0%) were classified as euthyroid, 136 (1.5%) as SCHyper, and 511 (5.5%) as SCHypo. Compared to euthyroid subjects, SCHyper participants presented significantly higher heart rate (68.8 *vs* 66.5 for euthyroidism, P=0.007) and shorter R-R intervals (871.4 *vs* 901.6, P=0.007). Although SCHyper was associated with lower standard deviation of NN interval (SDNN) (β: –0.070; 95% confidence interval (95%CI): –0.014 to –0.009) and low-frequency (LF) (β: –0.242, 95%CI: –0.426 to –0.058) compared to the euthyroid group, these differences lost significance after multivariate adjustment for confounders. No significant differences were found for HRV in SCHypo. No association was found between HRV and SCHyper or SCHypo compared to euthyroid subjects in this sample of apparently healthy subjects.

## Introduction

Since cardiovascular disease is the leading cause of mortality in the world (1), the identification of its risk factors is imperative. As the cardiovascular system is a major target for thyroid hormone action (2), both clinical and subclinical thyroid diseases are associated with several impairments, such as coronary heart disease (3–5), heart failure (6,7), increased risk of atrial fibrillation (8), and cardiovascular mortality (4).

In patients with thyroid dysfunctions, autonomic disturbances are observed (9), however, these impairments have been more often described for overt thyroid diseases, and the results of previous studies concerning the presence of similar alterations in subclinical diseases are still a matter of debate. Subclinical thyroid alterations appear to be an intermediate state between euthyroidism and overt dysfunctions; therefore, a deeper exploring of underlying mechanisms involved in cardiovascular conditions in subclinical thyroid disease is important for early diagnosis or prevention in the course of cardiovascular diseases.

The heart rate variability (HRV) analysis is a very useful and widely applied method for cardiac autonomic assessment. Although some heterogeneity exists, most studies on subclinical thyroid disorders reported an autonomic imbalance with lower HRV indices in both subclinical hyperthyroidism (SCHyper) (10–12) and hypothyroidism (SCHypo) (13–15). Evaluating the relationship between the subclinical dysfunctions and HRV may depict an important scenario regarding the underlying cardiovascular risk in this population.

The aim of the present study was to cross-sectionally evaluate the association between subclinical thyroid disorders and HRV time and frequency indices using baseline data of the Brazilian Longitudinal Study of Adult Health (ELSA-Brasil) (16 [Bibr B17]).

## Material and Methods

### Participants

This study is a cross-sectional analysis of the baseline data of ELSA-Brasil collected from August 2008 to December 2010. The ELSA-Brasil protocol, fully published elsewhere (16–19), included 15,105 civil servants, aged 35–74 years from six institutions of six Brazilian cities. The study aimed to determine the incidence of cardiovascular diseases and diabetes and their associated risk factors.

The sample of this study was composed by apparently healthy participants with a validated EKG recording. Therefore, subjects with previous cardiovascular impairments or surgery, that is, angina, cardiac revascularization, heart failure, myocardial infarction, and stroke, were excluded from the analysis. Participants under use of beta-blockers medications were excluded as well, due to the influence over the heart rate.

The protocol was approved at all six centers by the Institutional Review Boards addressing research in human participants according to Declaration of Helsinki. Written informed consent was obtained from all participants.

### Thyroid function

Thyroid-stimulating hormone (TSH) and free thyroxine (FT4) were measured by a third generation immunoenzymatic assay (Siemens, USA) in serum obtained from venous blood samples after an overnight fast and centrifugation 30 min after collection at 1500 *g* for 15 min at 5^o^C. FT4 levels were only evaluated in participants who presented altered TSH levels. In this study, SCHypo and SCHyper were classified in subjects reporting no use of medication for thyroid disorders treatment, TSH levels respectively above and under the reference range of 0.4 to 4.0 mIU/L, and FT4 concentration between 0.8–1.9 ng/dL (analytical sensitivity of 0.3 ng/dL), similar to those used in the National Health and Nutritional Examination Survey (NHANES III) (20) and recommended by Surks et al. (21).

As this study aimed to assess HRV in thyroid subclinical disorders, subjects with overt hypothyroidism or hyperthyroidism, those using drugs with thyroid effects or using any medication that could interfere with thyroid function, such as amiodarone, carbamazepine, carbidopa, phenytoin, furosemide, haloperidol, heparin, interferon, levodopa, lithium, metoclopramide, propranolol, primidone, rifampicin, and acid valproic were excluded.

### Heart rate variability

The protocol used to record and to analyze heart rate variability in ELSA-Brasil has been published elsewhere (22). Briefly, a 10-min resting-state electrocardiogram (EKG) recording was obtained in supine position during spontaneous breathing and without task demands. The EKG was always obtained in the morning (between 8:00 a.m. and noon) in a temperature-controlled room (21–24°C) and was sampled at 250 Hz with a digital electrocardiograph (Micromed, Brazil), consistent with international standards for the measurement of HRV (23). The WinCardio software (version 4.4a, Micromed, Brazil) was used to generate the R-R interval series from a selected lead (usually Lead II) associated with higher R-wave amplitude. The artifact detection and spectral analytic techniques were the same as used by Dantas et al. (24), in which the R-R series were automatically preprocessed to remove ectopic beats and artifacts, and linear interpolation was employed to replace any removed beats.

HRV analyses were performed both in time and in frequency domains. Time domain analysis included mean heart rate (in beats per minute, bpm), the standard deviation of NN interval (SDNN, ms), the percentage of adjacent NN intervals differing by more than 50 ms (pNN50, %), and the square root of the mean of the sum of the squares of differences between adjacent NN intervals (RMSSD, ms). Power spectral analysis was carried out by autoregressive modeling, estimated by the Yule Walker method, using the recursive algorithm of Levinson-Durbin and the high-frequency (HF) (0.15–0.40 Hz) and low-frequency (LF) (0.04–0.1 Hz) were calculated.

### Covariates

Each participant underwent an interview conducted by trained personnel with a strict quality control at their workplace, and a visit to the Research Center for clinical exams according to standard protocols (18). Questionnaires addressed age, gender, self-reported smoking status (classified as never, past, or current smoker based on a detailed description of lifetime tobacco use). Alcohol consumption was categorized as never, past, or current based on detailed lifetime information of alcohol consumption.

All prescription and over-the-counter pill bottles were reviewed for medications taken for the prior 15-day period. Height and weight were measured using light clothes and body-mass index was calculated as weight divided by squared height in meters. Blood pressure (BP) measurements were taken using the validated oscillometric device HEM 705CPINT (Omron, Japan). Three measurements were taken at one-minute intervals. The mean of the two latest BP measurements was considered as the clinical BP.

In this cross-sectional analysis, we defined hypertension as use of medication to treat hypertension, or a systolic blood pressure ≥140 mmHg, or diastolic blood pressure ≥90 mmHg (22). Diabetes was defined as previous medical history of diabetes, use of medication to treat diabetes, a fasting plasma glucose ≥126 mg/dL, a 2-hour plasma glucose ≥200 mg/dL in the glucose tolerance test, or an HbA_1C_ ≥6.5%. Dyslipidemia was defined as low density cholesterol ≥130 mg/dL or use of lowering cholesterol medications. Leisure physical activity was used according to the International Physical Activity Questionnaire (IPAQ) (25), which was categorized as low, moderate, and high.

### Statistical analysis

We expected a mean difference between groups around 10% with less than 40% standard deviation in the SDNN index, as commonly used in studies. By using the Minitab 18 software (Minitab, France), a power of 80% with an alpha of 0.05 was calculated for a sample size of 135 subjects (11,26-28).

Before statistical analyses, the normality was checked by the Shapiro-Wilk test and homogeneity of variances by the Levene test. We also assessed outliers by the boxplot method, using ‘fences’ of three interquartile ranges above and below the third and the first quartile, respectively, as limits for non-outlier observations (29). Continuous variables are reported as means ± standard deviation (SD) or median and interquartile range (IQR). SCHypo and SCHyper were compared with euthyroid subjects using Student’s *t*-test or Wilcoxon test, as appropriate after assessing normality assumptions. Categorical variables are reported as proportions and were compared using the chi-square test or Fisher’s exact test.

After natural logarithmic transformation of HRV variables for normalization, multivariate linear models were built using HRV as dependent variables and SCHypo or SCHyper in comparison with euthyroid subjects as independent variables. Data are reported as beta coefficients and 95%CI. Model 1 was adjusted for age, sex, and race. Model 2 was adjusted for variables in model 1 plus cardiovascular risk factors (body mass index, smoking status, hypertension, diabetes, dyslipidemia, binge drinking, and leisure physical activity).

Analyses were performed using SPSS 20.0 (IBM, USA). P<0.05 was used to indicate significance. All the tests were two-tailed.

## Results

**Figure 1:**
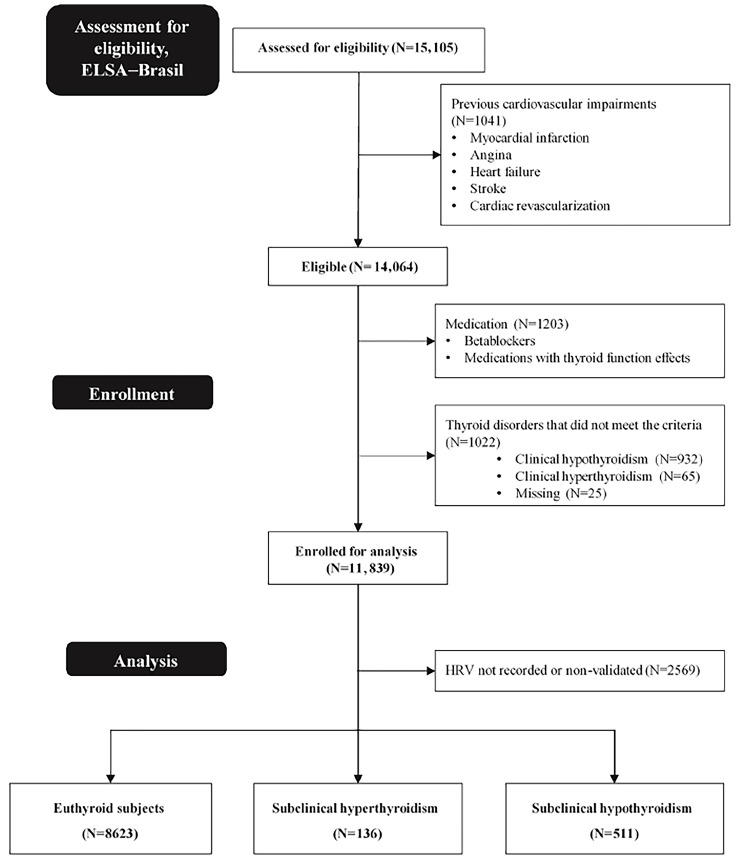
Flow-chart of the study. HRV: heart rate variability.

After exclusions (previous cardiovascular impairments: N=1041; medications: N=1203; thyroid disorders that did not meet the criteria: N=1022; HRV not recorded or non-validated: N=2569), a total of 9270 subjects were included, with median age of 50 years (IQR: 44–56 years); 8623 (93.0%) were classified as euthyroid, 136 (1.5%) as SCHyper, and 511 (5.5%) as SCHypo (Figure 1).

Table 1 shows that the SCHyper group had a higher frequency of women, a lower frequency of whites and higher of blacks, and more current smokers compared to euthyroid subjects. The SCHypo group had older participants, more whites and less blacks, less current smokers, and with higher BMI than the euthyroid group.

**Table 1.** General characteristics of the sample according to the presence of subclinical thyroid dysfunction.

	Euthyroidism (N=8623)	Subclinical hyperthyroidism (N=136)	P value*	Subclinical hypothyroidism (N=511)	P value*
Age (years)†	50.0 [44.0–56.0]	51.0 [44.0–57.0]	0.330	52.0 [46.0–59.0]	<0.001
Women (n, %)	4451 (51.6%)	85 (62.5%)	0.015	270 (52.8%)	0.624
Race (n, %)			0.046		<0.001
White	4230 (49.7%)	55 (41.4%)		296 (58.6%)	
Brown	2582 (30.3%)	39 (29.3%)		148 (29.3%)	
Black	1405 (16.5%)	34 (25.6%)		44 (8.7%)	
Other	302 (3.5%)	5 (3.8%)		17 (3.4%)	
BMI (kg/m^2^)†	26.1 [23.5–29.2]	25.7 [23.2–29.1]	0.445	26.5 [24.0–29.8]	0.014
Smoking (n, %)			0.001		<0.001
Never	5102 (59.2%)	62 (45.6%)		297 (58.1%)	
Past	2383 (27.6%)	44 (32.4%)		171 (33.5%)	
Current	1137 (13.2%)	30 (22.1%)		43 (8.4%)	
Hypertension (n, %)	2366 (27.4%)	44 (32.4%)	0.240	145 (28.4%)	0.683
Diabetes mellitus (n, %)	1381 (16.0%)	22 (16.2%)	1.000	74 (14.5%)	0.389
Dyslipidemia (n, %)^#^	4789 (55.6%)	73 (53.7%)	0.727	299 (58.5%)	0.206
Physical activity (n, %)			0.741		0.624
Low	6504 (76.4%)	105 (78.4%)		393 (78.1%)	
Moderate	1352 (15.9%)	21 (15.7%)		72 (14.3%)	
High	658 (7.7%)	8 (6.0%)		38 (7.6%)	
Alcohol consumption (n, %)					
Never	855 (9.9)	13 (9.6)	0.39	60 (11.7	0.89
Past	1579 (18.3)	27 (19.9)		89 (17.4)	
Current	6182 (71.8)	96 (70.6)		362 (70.8)	
TSH (mIU/L)†	1.5 [1.0–2.1]	0.3 [0.1–0.3]	<0.001	5.1 [4.4–6.4]	<0.001

†Median and interquartile range (IQR); BMI: body mass index; TSH: thyroid stimulating hormone; ^#^defined as homeostatic model assessment of insulin resistance (HOMA-IR) >75th percentile; *P values for comparison with euthyroid group (Student’s *t*-test or Wilcoxon test; chi-squared test for categorical variables).

As can be observed in Table 2, the median values of HRV variables indicate that SCHyper subjects presented higher heart rate, shorter R-R intervals, lower NN variance, and lower LF values in comparison with euthyroid subjects. In SCHypo, no differences were found in comparison to the euthyroid group.

**Table 2.** Median and interquartile range of the characteristics associated with heart rate variability according to the presence of subclinical thyroid dysfunction.

	Euthyroidism (N=8623)	Subclinical hyperthyroidism (N=136)	P value*	Subclinical hypothyroidism (N=511)	P value*
Mean HR (bpm)	66.5 [61.1–72.7]	68.8 [63.2–75.7]	0.007	66.2 [60.8–73.2]	0.769
Mean RR (ms)	901.6 [825.4–982.7]	871.4 [792.6–949.7]	0.007	906.9 [819.6–987.4]	0.769
SDNN (ms)	38.9 [29.7–50.4]	35.3 [27.5–48.3]	0.038	38.0 [29.7–48.2]	0.164
pNN50 (ms)	25.8 [18.0–36.2]	23.0 [16.0–35.5]	0.173	25.6 [17.5–34.3]	0.165
RMSSD (ms)	4.0 [0.7–13.2]	2.3 [0.3–13.0]	0.095	3.6 [0.7–11.1]	0.278
LF (ms^2^)	260.0 [124.7–513.7]	210.3 [96.6–456.2]	0.016	249.7 [122.7–467.8]	0.188
HF (ms^2^)	229.7 [106.1–484.3]	207.2 [92.7–456.3]	0.438	219.0 [96.5–420.5]	0.079

HR: heart rate; RR: R-R intervals; SDNN: standard deviation of NN interval; pNN50: percentage of adjacent NN intervals differing by more than 50 ms; RMSSD: square root of the mean of the sum of the squares of differences between adjacent NN intervals; LF: low-frequency component; HF: high-frequency component. *P values for comparison with the euthyroid group (Wilcoxon test).

The multivariate linear analysis shows negative association of SCHyper with ln(SDNN) (β=–0.077, 95%CI=–0.144 to –0.009, P=0.026) and ln(LF) (β=–0.242, 95%CI=–0.426 to –0.058, P=0.010) in crude analysis (Table 3). After adjustments for sex, age, and race, the association with ln(LF) was maintained (β=–0.177, 95%CI=–0.353 to –0.002, P=0.048), but it lost significance after adjustments for cardiovascular risk factors. In the SCHypo group, no association with HRV indices was verified for any model.

**Table 3.** Beta coefficients and 95% confidence intervals of multivariate linear regression models, evaluating the association between heart rate variability and subclinical hyperthyroidism (SCHyper) or subclinical hypothyroidism (SCHypo) versus euthyroid subjects.

	Subclinical hyperthyroidism and euthyroid (N=8759)		
	Crude	Model 1	Model 2
ln(SDNN) (ms)	–0.077 (–0.144 to –0.009; P=0.026)	–0.059 (–0.125 to 0.007; P=0.080)	–0.056 (–0.122 to 0.010; P=0.095)
ln(RMSSD) (ms)	–0.070 (–0.160 to 0.020; P=0.127)	–0.075 (–0.163 to 0.013; P=0.097)	–0.079 (–0.167 to 0.008; P=0.076)
ln(LF) (ms^2^)	–0.242 (–0.426 to –0.058; P=0.010)	–0.177 (–0.353 to –0.002; P=0.048)	–0.170 (–0.345 to 0.005; P=0.057)
ln(HF) (ms^2^)	–0.088 (–0.284 to 0.108; P=0.378)	–0.098 (–0.288 to 0.092; P=0.314)	–0.113 (–0.303 to 0.077; P=0.243)
	Subclinical hypothyroidism and euthyroid (N=9134)		
	Crude	Model 1	Model 2
ln(SDNN) (ms)	–0.028 (–0.064 to 0.007; P=0.121)	–0.004 (–0.039 to 0.030; P=0.804)	–0.006 (–0.040 to 0.029; P=0.749)
ln(RMSSD) (ms)	–0.034 (–0.081 to 0.014; P=0.166)	0.007 (–0.039 to 0.053; P=0.765)	0.005 (–0.041 to 0.051; P=0.834)
ln(LF) (ms^2^)	–0.083 (–0.180 to 0.014; P=0.094)	–0.002 (–0.094 to 0.090; P=0.965)	–0.001 (–0.093 to 0.092; P=0.991)
ln(HF) (ms^2^)	–0.088 (–0.191 to 0.015; P=0.094)	0.007 (–0.093 to 0.107; P=0.891)	0.000 (–0.099 to 0.100; P=0.993)

Model 1 was adjusted for age, sex, and race. Model 2 was adjusted for variables in model 1 plus hypertension, dyslipidemia, diabetes, smoking status, BMI, use of alcohol, and leisure physical activity. lnSDNN: log-transformed standard deviation of NN interval; ln(RMSSD): log-transformed square root of the mean of the sum of the squares of differences between adjacent NN intervals; ln(HF): log-transformed high-frequency component; ln(LF): log-transformed low-frequency component.

## Discussion

Participants with subclinical hyperthyroidism showed increased mean heart rate and decreased mean R-R intervals, compared to the euthyroid group. Crude HRV analysis indicated autonomic imbalance as shown by significantly lower values of SDNN and LF. SDNN represents the total variation between heart beats, that is, the global variability, and LF reflects a complex, not easily discernible mix of sympathetic, parasympathetic, and other unidentified factors (30), and is associated with baroreflex sensitivity (31,32). Although differences in the indices pNN50, RMSSD, and HF were found with lower values suggesting a withdrawal of vagal input to the heart of SCHyper subjects, they were not statistically significant. Since SDNN and LF are strongly correlated (R=0.867), as well as RMSSD, pNN50, and HF (R>0.90) (33), our results seem to indicate a physiologically plausible scenario.

As it is well known, thyroid hormones play a determining role in maintaining cardiovascular homeostasis and their increased or diminished action on cardiac and vascular molecular pathways cause important alterations (34,35). Accordingly, previous studies with smaller sample sizes showed reduced variability in subjects with SCHyper compared to euthyroid ones (10,11,14,26). Goichot et al. (11) detected lower values for SDNN in SCHyper subjects (N=12) compared to controls (N=32). Galetta et al. (28) (SCHyper: N=30; controls: N= 30), Petretta et al. (10) (SCHyper: N=30; controls: N= 20), and Falcone et al. (14) (SCHyper: N=28; controls: N=170; all with coronary artery disease), besides lower SDNN, also detected reduced RMSSD and pNN50 values when comparing both groups. It is noteworthy that we did not find a marked cardiac parasympathetic withdrawal in SCHyper subjects; however, this group showed lower values in magnitude for parasympathetic indices in comparison to euthyroids.

In the frequency domain, we found a difference in LF values between euthyroids and SCHyper subjects, but no difference in HF, as was reported by Petretta et al. (10). Since LF band of spectral analysis has been strongly associated with baroreceptor reflex sensitivity (31,32), our results suggested that, besides autonomic activity impairments, SCHyper subjects might present some additional baroreflex impairment. Portella et al. (12) also did not find association in HF index in 16 patients with SCHyper compared to 16 controls.

Studies with overt SCHyper subjects describe altered balance of the autonomous nervous system, characterized by both diminished vagal tone and enhanced sympathetic activity compared to controls (10,36–38). The parasympathetic inhibition might be due to action of thyroid hormone on central nervous system structures integrating autonomic functions (36), while an increased adrenergic responsiveness may represent the primary effects of the abnormal concentrations of thyroid hormones (10).

As the parasympathetic tone did not show profound impairment, observed by the vagal-related HRV indices, it seems that the effects of SCHyper on the autonomic nervous system are milder in relation to overt hyperthyroidism. Therefore, our results are in agreement with the assumptions of Goichot et al. (11), who suggests that subclinical hyperthyroidism is an intermediate state between euthyroidism and overt hyperthyroidism, and a continuum cardiovascular modification is related to the degree of thyroid hormone excess. According to Celik et al. (27), HRV alterations may represent an early phase of important autonomic dysfunction, characterizing a useful tool to monitor cardiovascular risk with important clinical implications, and support the decision of treatment.

Despite the verified association between SCHyper and SDNN and LF indices, in both group comparisons and the crude analysis of multivariate regression, we can see that this significance is lost after adjustments for the most important anthropometric and sociodemographic characteristics, as well as risk factors such as age, sex, race, body mass index, smoking status, alcohol consumption, hypertension, diabetes, dyslipidemia, and leisure physical activity. This means that these characteristics are determinants for the dependence relationship between global variability and low TSH levels and, sometimes, wrong conclusions can be assumed if they are simply disregarded.

Regarding the subclinical hypothyroidism group, no significant difference was verified compared to the euthyroid group. Our results diverge from previous findings, which described parasympathetic inhibition and global variability reduction in SCHypo. Galetta et al. (28), comparing 42 SCHypo subjects with 30 controls, found lower HF values in frequency domain analysis and reduced time domain indices. Falcone et al. (14) evaluated 55 SCHyper subjects compared to 170 euthyroids and observed lower SDNN and RMSSD.

In the study of Sahin et al. (15), no differences in LF and HF were found, whereas decreased SDNN was reported in the subgroup with TSH levels equal to or higher than 10 mIU/mL (N=13; 41.9% of total subclinical hypothyroidism group). In our sample, most cases of SCHypo were mild cases, with only 25 (0.27%) subjects presenting TSH levels higher than 10 mIU/L. High TSH and even subclinical hypothyroidism is associated with a higher risk of sudden cardiac death, compared with normal TSH levels (39), and a reduced HRV may be an early phase of myocardial impairment in patients with SCHypo (27). Thus, HRV analysis may represent a useful tool in monitoring the cardiovascular risk in these patients.

In the SCHypo group, no association with HRV indices was verified for any model, different from the findings of Celik et al. (27) and Galetta et al. (28), who found moderate linear correlation between high TSH levels and SDNN (R=–0.69 and R=–0.42, respectively). This divergence between our and previous results is possibly due to the values of TSH found in the sample, which are not much higher than the reference range.

Langén et al. (39) showed that baseline TSH, categorized in three levels (low: <0.4 mU/L; normal: 0.4–3.4 mU/L; and high: >3.4 mU/L), had a U-shaped association with the risk of total mortality. The findings of Chaker et al. (35) also suggested a U-shaped relation between thyroid function and the risk of sudden cardiac death. Comparatively, our results also suggested that SCHyper, euthyroidism, and SCHypo, respectively, show an inverted U-shaped association with HRV linear measures.

### Final considerations and conclusion

These results must be considered within the context of the study design. This is a cross-sectional analysis that can evaluate association, but not causality. At this moment, only a cross-sectional analysis is possible; however, in the near future, prospective data will be available and causality relationships may be delineated.

One important characteristic of this research is the magnitude of ELSA-Brasil study, which enrolled a total of 15,105 apparently healthy participants. The HRV was analyzed in 647 participants with subclinical thyroid dysfunctions (mean age around 53 years), which is a much larger sample size than any other study. The large number of subjects makes this sample more similar to the country population than to a selected clinical group. Additionally, to our knowledge, no other study analyzed the correlation between subclinical thyroid dysfunctions and HRV variables with adjustments for sociodemographic and clinical characteristics. Possible explanations for our negative results after multivariate adjustment may include the low levels of TSH in the sample and the mean age of the cohort around 52 years.

In conclusion, our results showed that SCHyper subjects presented lower global heart rate variability and the subclinical thyroid dysfunctions presented no relationship with HRV variables.

## Conflicts of interests

ÉJFPM is a medical manager at Bayer Pharmaceuticals AG, Brazil.
